# Early clinical outcomes of Naton robotic-assisted medial unicompartmental knee arthroplasty

**DOI:** 10.1007/s00264-025-06519-y

**Published:** 2025-04-16

**Authors:** Ke He, Yu Leng, Xiaoyi Jiao, Yuan Li, Qingshan Li, Guanjun Sun, Yi Yin, Xu Peng, Ke He’s

**Affiliations:** Department of Joint and Sports Medicine, Suining Central Hospital, 127 Dongping North Road, Chuanshan District, Suining City, Sichuan Province China

**Keywords:** Osteoarthritis, Robot-assisted, Arthroplasty, Clinical outcomes

## Abstract

**Purpose:**

Unicompartmental Knee Arthroplasty (UKA) has garnered increasing attention in recent years. Robotic-assisted systems have demonstrated enhanced precision, contributing to improved patient survival rates, satisfaction, soft-tissue balancing, alignment, and component sizing. The purpose of this study is to evaluate the early clinical outcomes of Naton robotic-assisted medial UKA by analyzing postoperative radiographic positioning of the unicompartmental prosthesis and comparing preoperative and postoperative functional outcomes in patients.

**Methods:**

A retrospective analysis was conducted on the clinical data of 32 patients (32 knees) who underwent Naton robotic-assisted medial UKA at Suining Central Hospital of Sichuan Province from November 2023 to January 2024. The cohort included ten males and 22 females, with a mean age of 70.53 ± 8.08 years, ranging from 53 to 88 years. All patients underwent surgery using the Naton robotic system and the Zhengtian Unique fixed-bearing UKA prosthesis. Radiographic (X-ray) findings, knee function, and complications were evaluated during follow-up. Radiographic assessments included prosthesis position, angle deviation, and posterior tibial slope (PTS). Knee function was assessed using a range of motion (ROM), Knee Society Score (KSS), Oxford Knee Score (OKS), and Forgotten Joint Score (FJS).

**Results:**

All patients in the study were followed for a period of eight to ten months, with a mean follow-up of (9.16 ± 0.68) months. No complications such as poor incision healing, periprosthetic infection, periprosthetic fracture, or prosthesis loosening were observed during the follow-up period. The medial unicondylar prostheses were found to be in place in all 32 cases, and no abnormal deviation of the prosthesis implantation angle was observed compared to immediate postoperative radiographs. The posterior tibial slope (PTS) was reduced from 13.00 ± 2.72° preoperatively to 5.08 ± 1.14° postoperatively, with a statistically significant difference (*P* ≤ 0.05). At the final follow-up, the knee range of motion (ROM) was improved from 107.03 ± 11.69° preoperatively to 128.25 ± 16.52° postoperatively. The KSS was improved from 46.28 ± 7.27 to 82.34 ± 14.72, and the OKS was improved from 36.13 ± 4.71 to 15.78 ± 3.52, all with statistically significant differences compared to preoperative values (*P* ≤ 0.05). The Forgotten Joint Score (FJS) was recorded as 89.2 ± 2.9.

**Conclusions:**

The short-term follow-up indicates a favorable prosthesis in situ rate for unicompartmental knee arthroplasty assisted by the Naton robot, with satisfactory knee function and patient-reported outcomes. The short-term clinical outcomes are satisfactory.

## Introduction

Unicompartmental knee arthroplasty (UKA) is recognized as a relatively minimally invasive knee replacement procedure that offers advantages such as reduced surgical trauma, less blood loss, faster recovery, maximal preservation of knee bone stock, and improved postoperative joint function [1–4]. However, it is difficult to achieve a personalized and precise surgery when performing UKA using traditional techniques and instruments. Patients may experience inaccuracies in osteotomy thickness and prosthesis placement, resulting in early aseptic loosening of the implant, lower long-term survival rates compared to total knee arthroplasty, and consequently higher rates of revision surgery [5–7]. Studies have indicated that the rate of non-standard prosthesis implantation in conventional UKA surgery can reach as high as 30%. Therefore, achieving precise osteotomy and accurate placement of the UKA prosthesis has become a critical focus in unicompartmental knee arthroplasty [8].

Robotic technology has demonstrated significant advances in surgical procedures. Particularly in orthopaedics, its application has gradually increased and has been shown to yield satisfactory clinical outcomes compared to traditional surgery. Robot-assisted unicompartmental knee arthroplasty is performed after preoperative CT planning of the hip, knee, and ankle is completed and adapted to intraoperative conditions, using computer and robotic arm technology to achieve precise osteotomy of the knee joint and accurate placement of the unicompartmental prosthesis [9–14]. Osteotomy and prosthesis placement in traditional UKA is more dependent on operator experience and standardized procedures. In contrast to the traditional manual technique, with the continuous development of robotic technology, repeatable and precise intraoperative positioning and placement of the prosthesis have gradually been achieved, minimizing the interference of human factors [15, 16].

China’s self-developed knee replacement robot technology started late, previous research was dominated by foreign production robots, and the effect of unicondylar arthroplasty by domestic robots has not been reported. The Naton Surgical Robot is a joint replacement assistive robot developed and produced by China’s Naton Science and Technology Group and marketed in 2023. Which has the following features: (1) it can realize different dimensional cuts of the bone surface in the form of points, lines, and surface, (2) mechanisms such as a safety wall, power failure protection, and visual intuitive feedback are established to ensure surgical safety, (3) the ability to implement full-process dynamic soft tissue gap assessment during surgery and fine-tuning based on the operator’s experience, (4) the ability to adapt to most Chinese and foreign prostheses at a later stage, and (5) a relatively low price. In 2023, the Department of Joint and Sports Medicine at Suining Central Hospital in Sichuan Province began utilizing the domestically produced Naton surgical robot to assist in UKA. A total of 32 robotic-assisted surgeries were successfully performed, with all patients receiving regular follow-up. The aim of this study is to evaluate the short-term clinical outcomes of Naton robotic-assisted medial UKA.

## Materials and methods

### Study cohort

Inclusion criteria: ① Patients must meet the latest diagnostic criteria for medial compartment osteoarthritis of the knee, with pain localized to the medial compartment confirmed by imaging and physical examination; ② Indications for medial unicompartmental knee arthroplasty (UKA) must be present, specifically a varus deformity primarily within the joint of less than 15°, with no significant extra-articular deformities. In addition, knee flexion contracture must be less than 15°, and range of motion must be at least 90°. Varus stress radiographs must be correctable to a neutral position, and the integrity of the lateral compartment cartilage must be confirmed through preoperative magnetic resonance imaging (MRI) and valgus stress radiographs. The medial and lateral collateral ligaments and the anterior and posterior cruciate ligaments must be functional; ③ Kellgren-Lawrence grade III or IV; ④ The patellofemoral joint and lateral compartment should be normal or show mild degeneration without associated symptoms. There should be no severe osteoporosis or bone destruction, and the surrounding soft tissues must be satisfactory; ⑤ Patients and their families must provide informed consent for study participation and all data must be complete; ⑥ Patients should have no prior surgical history of the knee joint, no severe mental illness or cognitive dysfunction, and must demonstrate good compliance with postoperative rehabilitation exercises. Exclusion criteria: ① Patients with severe knee deformity, knee stiffness, inflammatory or autoimmune joint disease; those with lateral compartment osteoarthritis; or severe patellofemoral arthritis with subluxation; ② Patients who underwent conversion to total knee arthroplasty during the procedure; ③ Patients with severe cardiovascular, hepatic, renal, or malignant disease contraindicating surgery; ④ Patients with acute or chronic infection of the knee or other body parts; ⑤ Follow-up duration of less than eight months; ⑥ Patients Incomplete clinical or radiographic data; ⑦The occurrence of major diseases (such as tumours, cardiovascular and cerebrovascular events, etc.) during the follow-up period significantly impacts the patient’s physical condition and the assessment of knee function. This retrospective analysis included 32 patients (32 knees) who underwent robot-assisted unicompartmental medial knee arthroplasty at Suining Central Hospital from November 2023 to March 2024. The cohort included ten males and 22 females ranging in age from 53 to 88 years, with a mean age of (70.53 ± 8.08) years. Body mass index (BMI) ranged from 19.5 to 30.0 kg/m², with a mean of (25.25 ± 4.3) kg/m²(Table 1). This study was approved by the Ethics Committee of Suining Central Hospital, Sichuan Province, China (Lun Audit No. S2023-80). All patients signed an informed consent for the use of robotic assistance.

**Table 1:** Basic characteristics

Variables	
Age (years)	70.53 (range 53–88, SD: 8.08)
Gender (male: female)	10 (10 R-UKAs): 22(22 R-UKAs)
Side of R-UKA (left: right)	14: 18
BMI (kg/m2)	25.25(range 19.5–30.0, SD: 4.3)
Operation time (min)	88 (range 52–125, SD: 20)
Hospital stays (days)	7.1 (range 5–9, SD: 2.1)
Follow-up (mos)	9.16(range 8–10, SD: 0.68)

## Preoperative planning

**Fig. 1:**
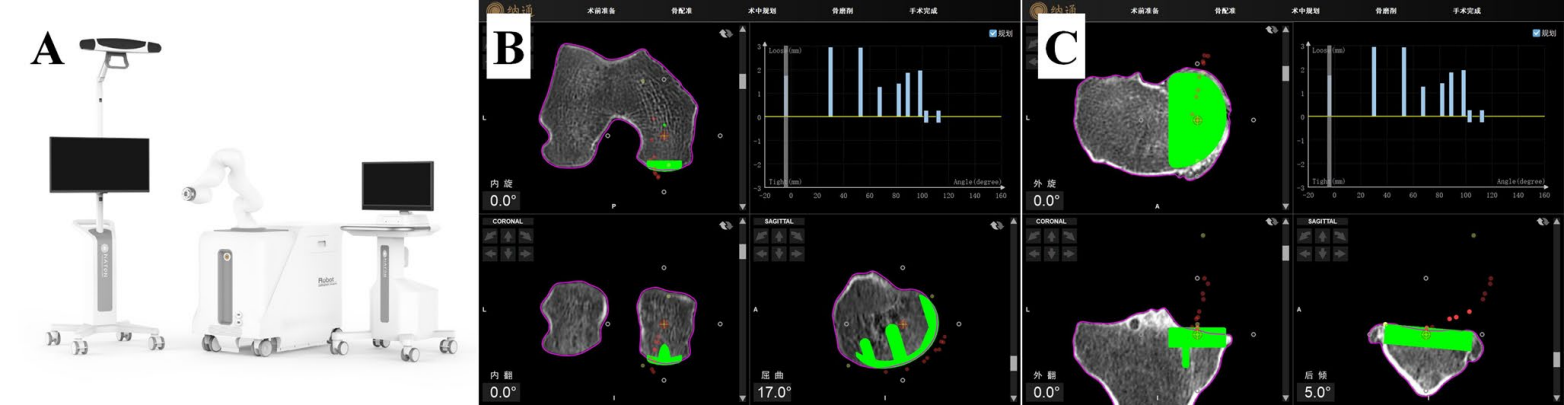
**A** The main structure of Naton surgical robot; **B** Intraoperative adjustment of femoral prosthesis; **B** Intraoperative adjustment of femoral prosthesis; **C** Intraoperative adjustment of tibial prosthesis

The surgical procedure is performed using the Naton robotic system (Naton Technology Group, China) (Fig. 1A). All surgeries are performed by a senior orthopaedic surgeon from our department who has completed specialized training in robotic surgery. The unicompartmental prosthesis used is the Zhengtian Unique fixed-bearing unicompartmental knee prosthesis (Naton Technology Group, China).

Preoperatively, all patients were required to undergo weight-bearing knee radiographs, varus and valgus stress radiographs, patellar axial radiographs, full-length lower extremity radiographs, and magnetic resonance imaging (MRI) of the knee. A comprehensive CT scan of the hip, knee, and ankle on the same side is performed to ensure a thorough evaluation, and the CT images are transferred to the robotic planning workstation for preoperative planning. The CT images are reconstructed by identifying anatomical landmarks to accurately determine both the anatomical and mechanical axes of the lower extremity. The 3D CT images are used in conjunction with the CT data to determine initial specifications for the types, sizes, positions, and models of femoral and tibial prostheses. Adjustments to prosthesis implantation angles and osteotomy thickness can be made in the sagittal, coronal, and rotational planes. The primary goal of preoperative planning is to restore the neutral force line of the lower extremity. The primary goal of preoperative planning was to approximate or restore the neutral mechanical axis of the lower limb, using the knee joint space and soft-tissue balance as references. During surgery, further fine-tuning of the prosthesis type and position can be made based on real-time imaging, data feedback, and the actual situation, ultimately confirming the surgical plan (Fig. 1B, C).

## Surgical procedures

**Fig. 2:**
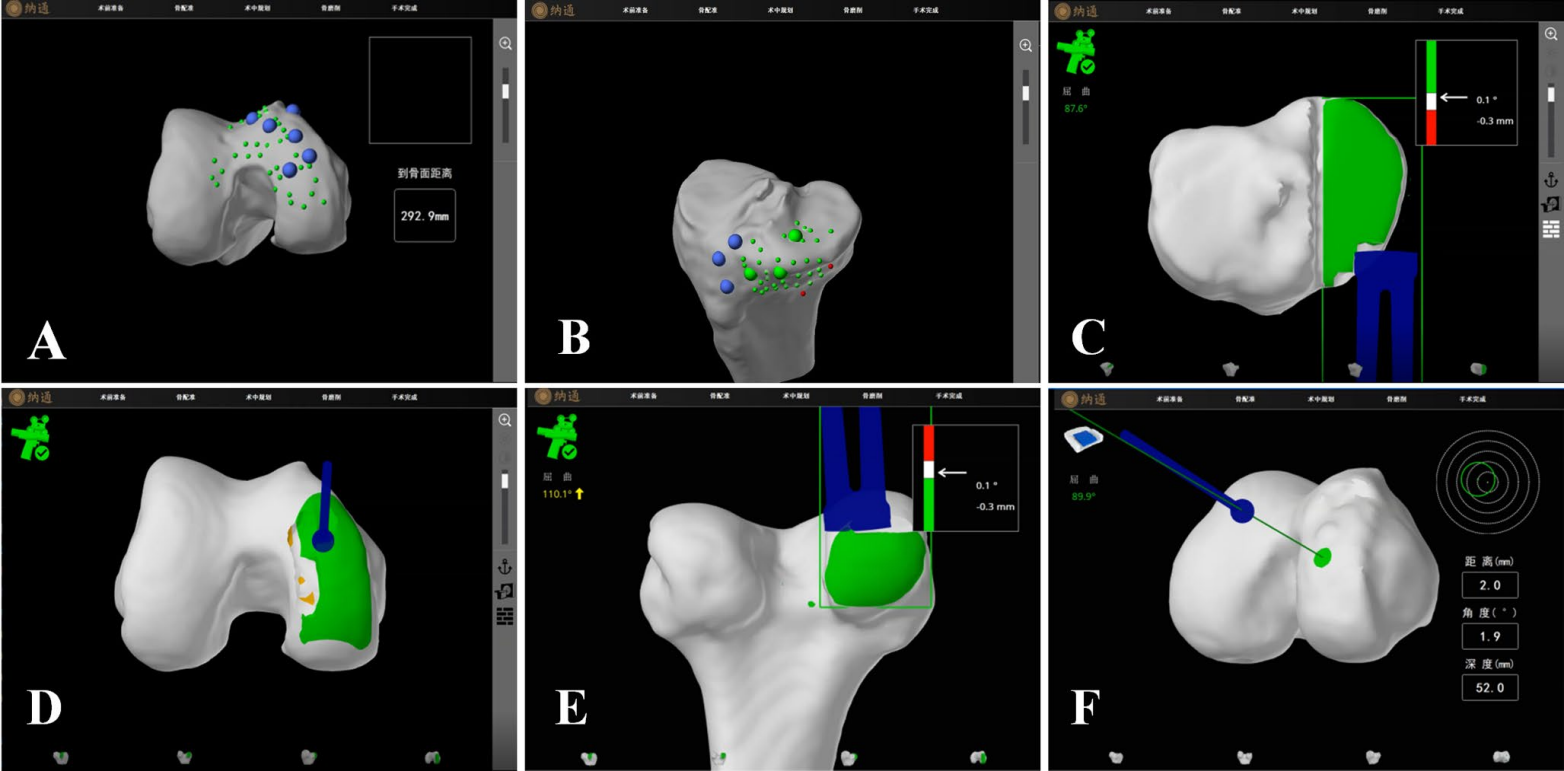
**A** Intraoperative calibration of the femoral side of the robot; **B** Intraoperative calibration of the tibial side of the robot; **C** Intraoperative tibial plateau horizontal osteotomy was performed; **D** Distal femoral osteotomy was performed during the operation; **E** The posterior condyle of femur was osteotomy during the operation **F** The central column of the femoral prosthesis was drilled

**Fig. 3:**
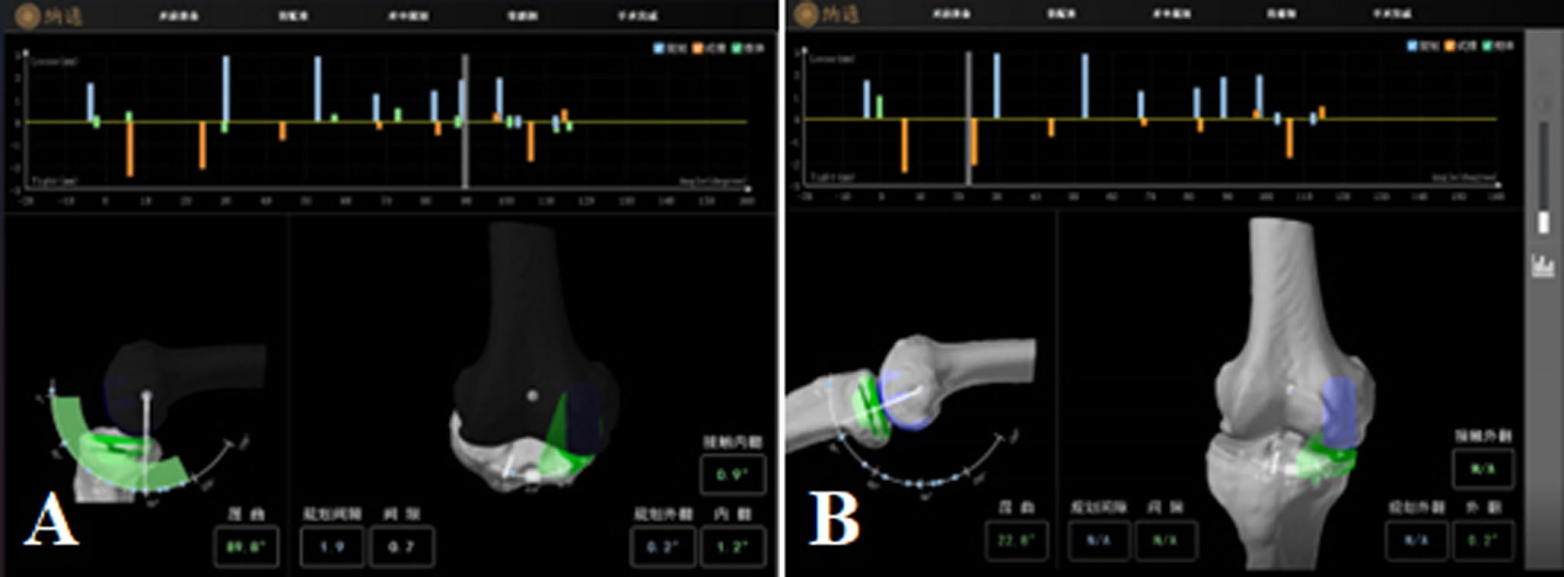
**A** Intraoperative knee space model measurement; **B** The knee space was measured after final prosthesis installation

**Fig. 4:**
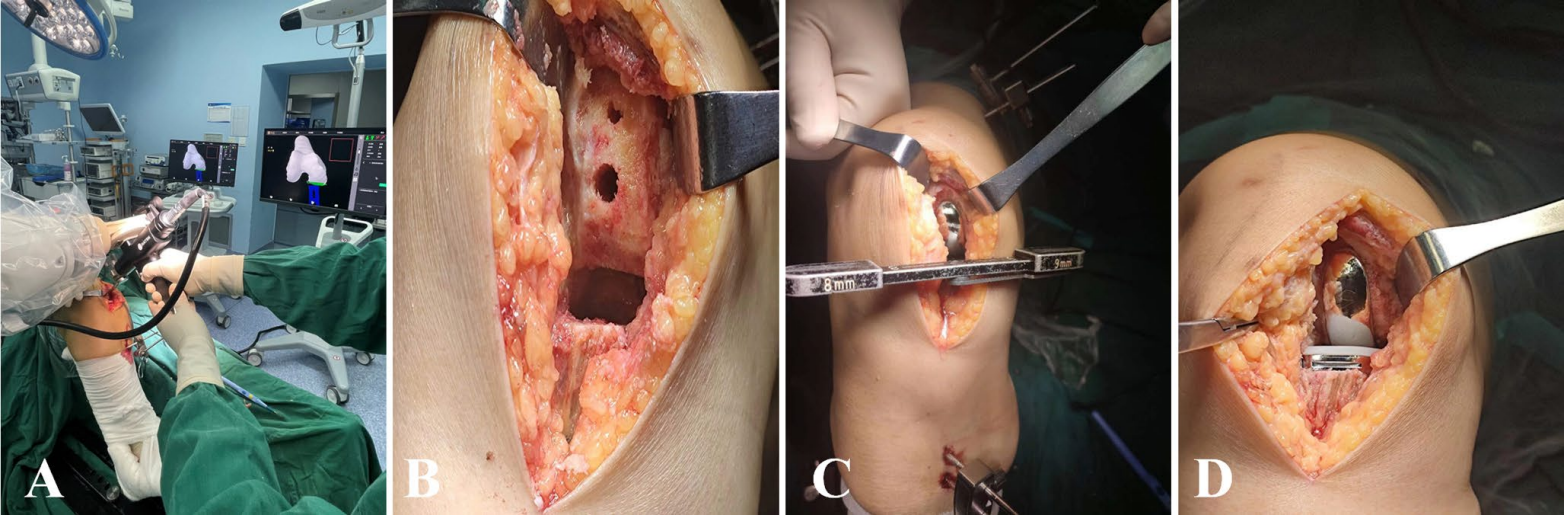
**A** Osteotomy with robot assistance; **B** Post-osteotomy image; **C** The space was measured intraoperatively; **D** The medial unicompartmental component was in good position

The patient is placed in the supine position under general anesthesia. The surgical site is disinfected and draped. A Naton ankle distractor is used to stabilize the surgical limb, along with lateral femoral support. A tourniquet is routinely applied. The incision begins at the superior medial border of the patella and terminates at the medial tibial tubercle, measuring approximately 8 cm. The incision is made through the skin, subcutaneous tissue, and fascia, layer by layer until the joint capsule is reached. The medial and lateral compartments of the knee joint, the patellofemoral joint, and the anterior cruciate ligament (ACL) are examined to confirm any isolated medial compartment pathology and the integrity of the ACL. If ACL rupture, patellofemoral groove changes, subluxation, or significant lateral compartment cartilage damage is confirmed, conversion to total knee arthroplasty is indicated. The procedure begins with knee flexion and partial excision of the infrapatellar fat pad. Two parallel positioning pins are placed above the femoral condyle and mid-tibia to which the femoral and tibial navigation frames are attached. Probes are used to confirm intraoperative localization (Fig. 2A, B). After bone spurs are removed, soft tissue balance is assessed. Prior to osteotomy, the amount of bone resection and prosthesis positioning were adjusted based on the flexion-extension gap of the knee joint and the trajectory of the implant. Typically, a 2–3 mm flexion-extension gap was reserved as a reference for fine-tuning the osteotomy volume and implant position to ensure optimal soft-tissue balance during knee flexion and extension. The tibial checkpoint was reverified, and the robotic system was positioned in the designated area adjacent to the operating table. Tibial osteotomy was then performed under real-time 3D navigation (Fig. 2C). Following this, the femoral checkpoint was validated, and femoral osteotomy was subsequently carried out. Real-time 3D navigation is used to complete drilling for the two femoral prosthesis fixation posts and to complete the osteotomy (Fig. 2D, E, F). Pulse irrigation is performed to cleanse the joint, the residual meniscus is resected, and a trial prosthesis is placed to confirm alignment with the preoperative plan. Once the prosthesis position is confirmed, a trial liner is placed. The stability of the joint, flexion-extension gap balance, and varus-valgus mobility are carefully assessed under navigation guidance (Fig. 3A). Once satisfied, the trial components are removed and another pulse lavage is performed. A “cocktail” analgesic is injected, and the femoral and tibial prostheses are placed and fixed with bone cement, followed by insertion of the liner. After the bone cement has been set, the flexion-extension gap, stability, and mobility of the knee joint are reassessed under navigation guidance (Fig. 3B). The joint cavity is irrigated again, a drain is placed, and the incision is closed layer by layer with great care (Fig. 4).

## Postoperative management

Postoperatively, antibiotics were routinely administered for 24 h. Patients began oral rivaroxaban six hours after surgery for thromboprophylaxis and, in the absence of contraindications, continued this medication until 21 days postoperatively. The drainage tube was removed on the first postoperative day. Non-steroidal anti-inflammatory drugs (NSAIDs) were prescribed for pain relief and were continued for two months post-surgery. On the first postoperative day, knee radiographs and lower extremity venous Doppler ultrasound were performed. Under the guidance of a rehabilitation physician, patients initiated exercises including ankle pumps, straight leg raises, and knee flexion-extension movements. Following discharge, patients continued personalized rehabilitation exercises under professional supervision.

## Clinical outcome measures

**Fig. 5:**
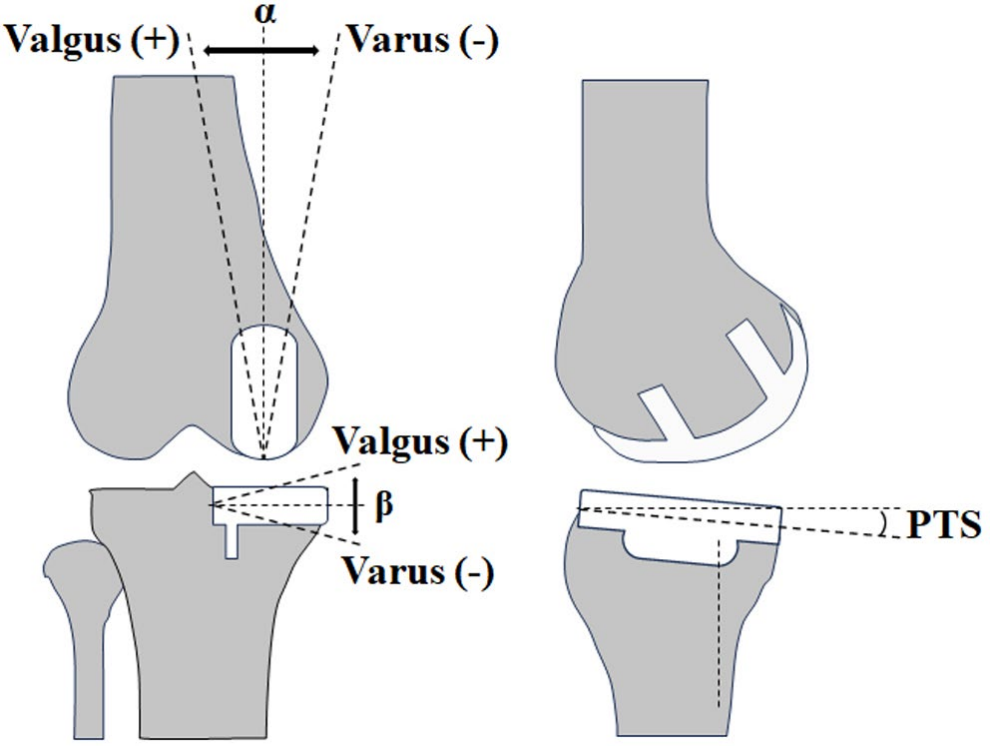
Schematic representation of the imaging measurements: α femoral component angulation; β tibial component angulation; PTS posterior tibial slope

Follow-up imaging and knee function assessments should be recorded at one month, three months, and final follow-up. Functional assessment indicators include Range of Motion (ROM) of the knee joint, Knee Society Scores (KSS), Oxford Knee Scores (OKS), and Forgotten Joint Score (FJS). The KSS (2011 edition) satisfaction scoring standard was used to score patients’ satisfaction (0–40 points), the higher the score, the more satisfied。Complications such as poor wound healing, wound infection, prosthesis-related infection, prosthesis loosening, and periprosthetic fractures should be monitored. Imaging studies should include weight-bearing radiographs of the knee joint and full-length radiographs of the lower extremity. Imaging measurements should be standardized for lower limb radiographs: orthopantomograms should be taken with the patient’s lower limbs upright, feet parallel, and toes pointing straight ahead to avoid rotation of the affected limb, and lateral radiographs should be taken with the medial and lateral condyles of the femur overlapping. All measurements were made manually by two senior physicians in the imaging department, and the average of the values measured by the two measurers was taken as the final result (Table 2), and all measurements were made using the measuring tools of the radiological imaging system (Infinitt pacs). The varus/valgus alignment angle of the knee prosthesis should be measured on the weight-bearing anteroposterior radiograph. To measure the femoral side prosthesis varus/valgus angle, measure the angle between the central axis of the femoral prosthesis and the mechanical axis of the femur. A valgus position is recorded as a positive value, while a varus position is recorded as a negative value. Tibial Side Prosthesis Varus/Valgus Angle: Measure the angle between the perpendicular to the coronal plane of the tibial prosthesis base and the tibial axis. A valgus position is recorded as a positive value, while a varus position is recorded as a negative value. On the weight-bearing lateral radiograph, record the posterior tibial plateau tilt angle both preoperatively and postoperatively for the tibial prosthesis (Fig. 5).

**Table 2:** Consistency analysis of imaging measurements

		ICC intragroup correlation coefficient	95%CI
Immedialy Postoperative (preoperative)	Femoral prosthesis measurement angles	0.926	0.855∼0.963
	Tibial prosthesis measurement angles	0.889	0.785∼0.944
	PTS	0.817	0.658∼0.906
Final follow-up	Femoral prosthesis measurement angles	0.83	0.680∼0.913
	Tibial prosthesis measurement angles	0.902	0.809∼0.951
	PTS	0.845	0.706∼0.921

### Statistical analysis

Statistical analyses were performed using SPSS 21.0 software (IBM Corporation, USA). The median, mean, quartiles, and interquartile range were used to describe the study sample. Measurement data were expressed as x ± s. Differences in preoperative and postoperative data between groups were compared using paired Student’s t-test, and all data for each cohort were verified as normally distributed using Shapiro-Wilk’s w-test. Differences were considered statistically significant at *P* < 0.05.

## Results

### Assessment of knee joint function

All patients in the study were followed for a period of eight to ten months, with a mean follow-up of (9.16 ± 0.68) months. No patient experienced poor wound healing or infection at the incision site. Range of motion (ROM) of the knee joint improved from (107.03 ± 11.69)° preoperatively to (128.25 ± 16.52)° at the final follow-up, a statistically significant difference (*P* < 0.01)(Table 3). The Knee Society Score (KSS) clinical score increased from (46.28 ± 7.27) points preoperatively to (82.34 ± 14.72) points at the final follow-up. The Oxford Knee Score (OKS) also improved significantly from (36.13 ± 4.71) points preoperatively to (15.78 ± 3.52) points at the final follow-up (*P* < 0.01). The Forgotten Joint Score (FJS) ranged from 76 to 95 points, with a mean of (80.78 ± 7.58) points. The average satisfaction score was 36.0 ± 1.5 (range, 30–38) indicating a high level of patient satisfaction (Table 4).

**Table 3:** Comparison of preoperative and postoperative PTS and ROM angles

Time	PTS(°)	ROM(°)
preoperative	13.00 ± 2.72	107.03 ± 11.69
final follow-up	5.08 ± 1.14	128.25 ± 16.52
t value	16.01	-11.09
P value	<0.01	<0.01

**Table 4:** Comparison of preoperative and postoperative KSS, OKS, FJS scores and patient satisfaction

Time	KSS scores	OKS scores	FJS scores	Patient satisfaction
preoperative	46.28 ± 7.27	36.13 ± 4.71	-	-
final follow-up	82.34 ± 14.72	15.78 ± 3.52	80.78 ± 7.58	34.8 ± 1.6
t value	-12.92	20.29	-	-
P value	<0.01	<0.01	-	-

### Radiologic evaluation

**Fig. 6:**
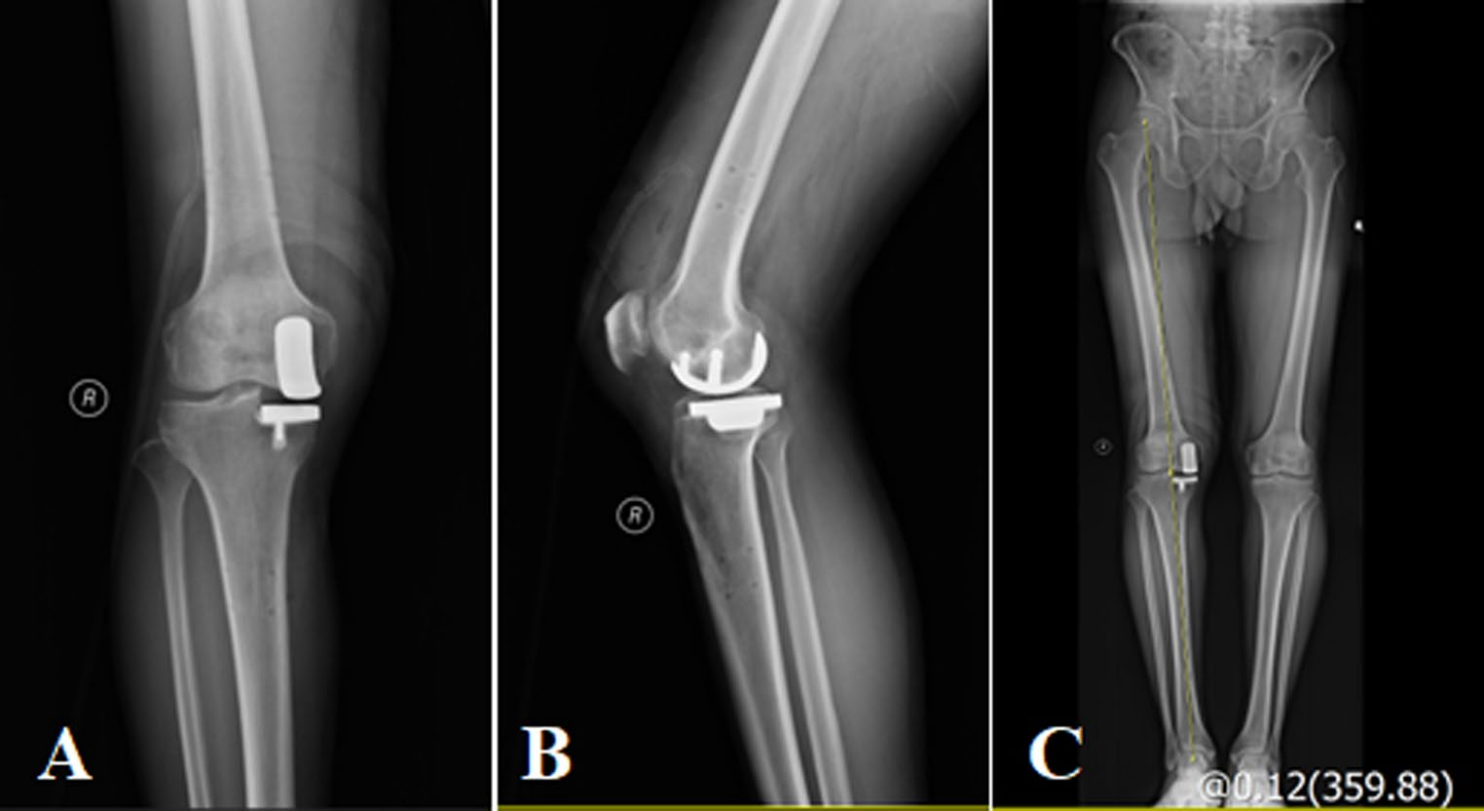
**A**, **B** Postoperative anterolateral radiographs of the right knee joint showed that the prosthesis was in good position; **C** Full-length radiographs of the lower extremity showed good lines of force in the right lower extremity

**Fig. 7:**
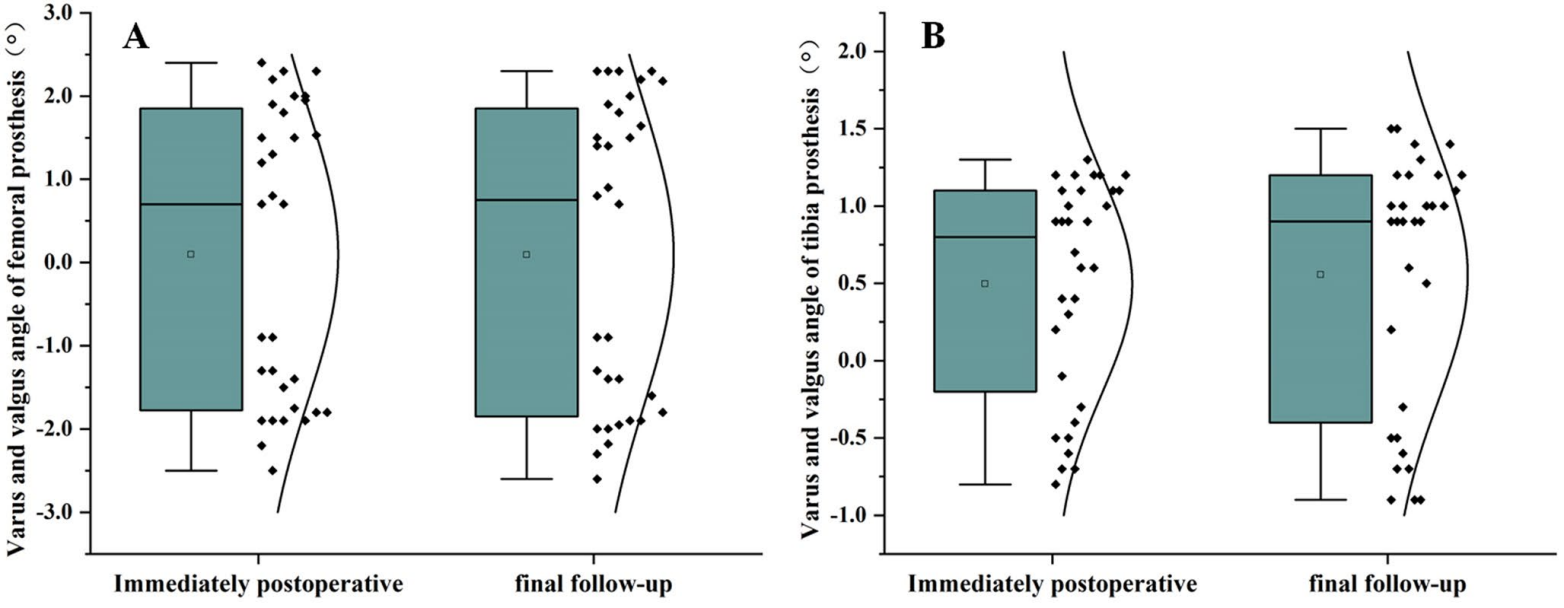
**A**, **B** Trends in the distribution of varus and valgus angles of femoral and tibial prostheses in the immediate postoperative period and final follow-up (*p* > 0.05)

**Fig. 8:**
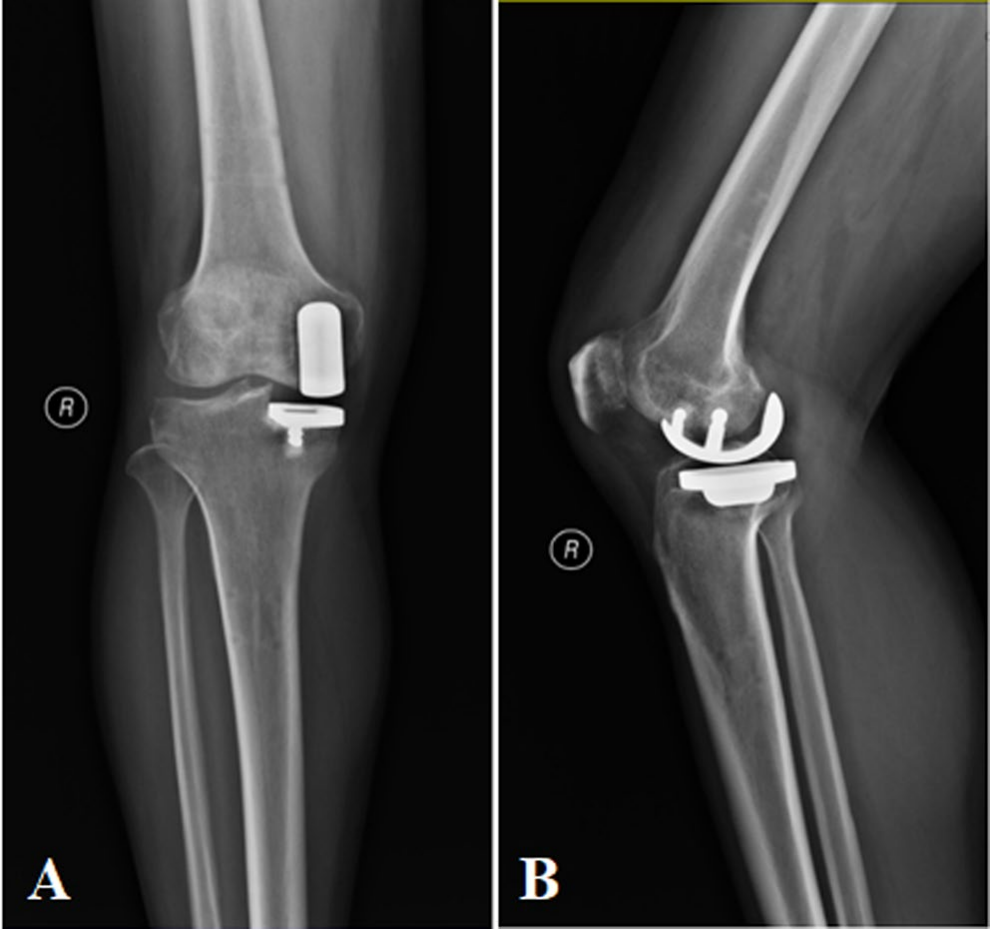
**A**, **B** The patient was followed up 8 months after surgery, and X-rays showed that the prosthesis was in good position without loosening

**Fig. 9:**
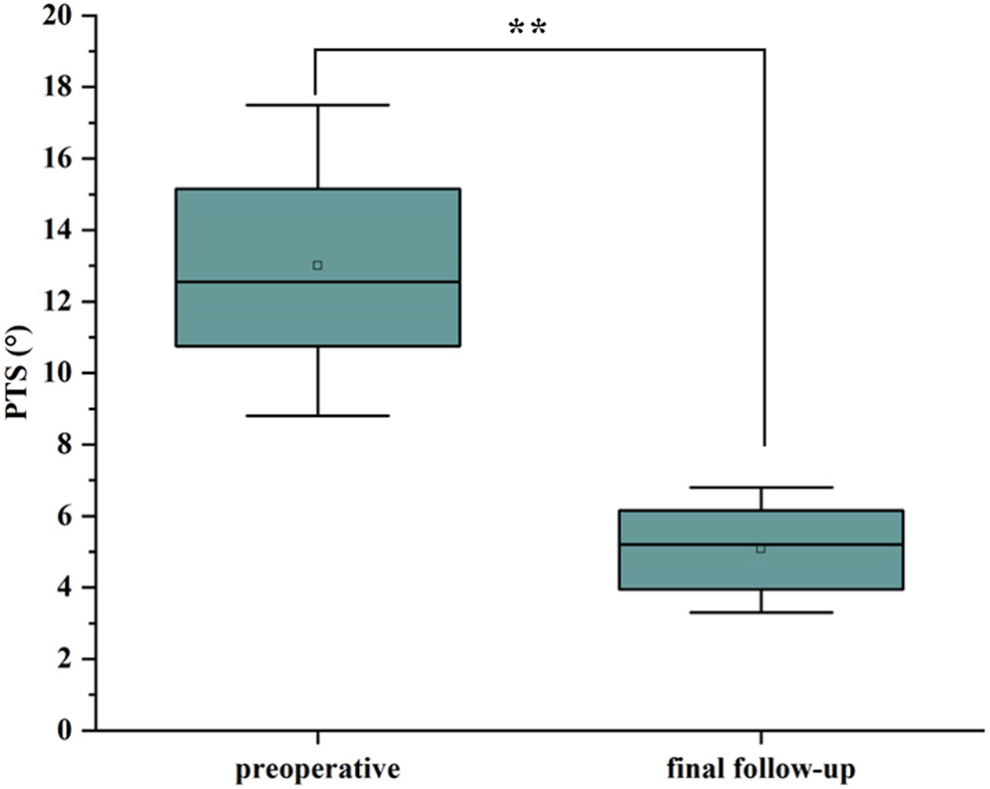
Distribution of PTS preoperatively and at final follow-up. PTS improved significantly after robotic unicondylar arthroplasty (*p* < 0.01)

All 32 patients had their single-compartment prostheses in place and the positioning was satisfactory (Fig. 6). No periprosthetic infections, prosthesis loosening, or periprosthetic fractures were observed. At the final follow-up, the coronal femoral component angulation ranged from − 2.60° to 2.30°, with a mean of (0.84 ± 1.77)°, showing no statistically significant difference compared to the immediate postoperative measurement of (0.77 ± 1.67)°(Fig. 7A). The tibial component angulation ranged from − 0.90° to 1.50°, with an average of (0.56 ± 0.83)° (Fig. 7B), which also showed no statistically significant difference compared to the immediate postoperative measurement of (0.50 ± 0.70)°(Table 5). Using 3° as the cutoff for abnormal values, no abnormalities were found in the positioning of the femoral and tibial components (Fig. 8). The Tposterior tibial slope (PTS) improved from a preoperative range of (8.8°-17.2°), mean (13.00 ± 2.72)°, to a postoperative range of (3.3°-6.5°), mean (5.08 ± 1.14)°(Fig. 9). The difference was statistically significant Table 3.

**Table 5:** Angle comparison of femoral and tibial prostheses in different postoperative periods

Time	Varus and valgus angle of femoral prosthesis(°)	Varus and valgus angle of tibia prosthesis(°)
Immediately postoperative	0.77 ± 1.67	0.50 ± 0.70
final follow-up	0.84 ± 1.77	0.56 ± 0.83
t value	-1.96	-1.48
P value	>0.05	>0.05

## Discussion

Unicompartmental knee arthroplasty has emerged as an effective method for treating end-stage medial compartment osteoarthritis because it preserves the surrounding anatomical structures of the knee joint and maintains the physiological characteristics of lower extremity motion [17, 18]. This procedure is characterized by rapid postoperative recovery and positive patient experiences. When performing unicompartmental knee arthroplasty (UKA), the primary consideration should be precision. Unlike total knee arthroplasty (TKA), UKA has a lower tolerance for error [19]. Improper implant positioning, malalignment, and ligament imbalance can all lead to UKA failure. Numerous studies have demonstrated that, compared with conventional techniques, robotic-assisted surgery significantly improves the accuracy of bone preparation, implant positioning, implant alignment, and soft-tissue balancing [20, 21]. Khow et al. [22]. conducted a follow-up study of 264 cases of medial unicompartmental arthroplasty with fixed-bearing prostheses to determine the effect of femoral component positioning on the longevity of these implants. They measured the coronal angles of the femoral and tibial components on postoperative radiographs and categorized those with varus or valgus angles greater than 3° as the OG group and those with angles less than 3° as the AG group. Follow-up examinations were performed at six months, two years, and ten years postoperatively. They found that both prosthesis lifetime and Oxford Knee Score (OKS) scores were lower in the OG group than in the AG group, concluding that in fixed-bearing medial unicompartmental arthroplasty, a femoral component varus/valgus angle greater than 3° significantly affects long-term efficacy. Diezi et al. [23] previously suggested that varus/valgus angles of the tibial component exceeding 5° would exacerbate wear on the tibial side of fixed bearings. Innocenti et al. performed a finite element analysis of the stress distribution of the UKA tibial component and polyethylene liner under 6° varus/valgus conditions. The results indicated that the lowest stress occurred when the tibial component was in a neutral position or 3° of valgus.

It is well known that precise bone resection and accurate implant positioning are critical to the clinical outcomes and long-term survival of unicompartmental knee arthroplasty (UKA). Intraoperative malpositioning of the prosthesis may lead to under- or over-correction of lower limb alignment, potentially resulting in early prosthetic loosening and the development of osteoarthritis in the contralateral compartment [24–27]. Previous studies have demonstrated that using a threshold of 3° deviation for implant alignment, the success rate of prosthetic placement in robotic-assisted UKA was 87%, compared to only 28% in the conventional surgery group [28]. The results of this study revealed that at the final follow-up, the range of coronal femoral component alignment deviation measured radiographically was − 2.60° to 2.30°, with a mean of (0.84 ± 1.77)°, while the tibial component alignment deviation ranged from − 0.90° to 1.50°, with a mean of (0.56 ± 0.83)°, all within the 3° threshold. Therefore, we have reason to believe that the NATON robotic system can effectively assist clinicians in achieving optimal positioning of the implanted prosthesis.

Posterior tibial slope (PTS) is also critical to the performance of the tibial component. The average PTS in normal adults is 8°. Excessive posterior tilt of the tibial prosthesis can increase stress on the posterior tibial bone, leading to ACL tears and prosthesis loosening [29]. Hernigou retrospectively analyzed 18 patients with anterior cruciate ligament deficiency who underwent unicompartmental arthroplasty with a follow-up of up to 16 years. In this study, none of the 11 patients with PTS less than 5° showed prosthetic loosening and all prostheses remained in place without revision. In contrast, the seven patients with a PTS greater than 8° required revision surgery. The authors also recommended that the PTS be less than 7° [30]. In this study, postoperative femoral component angles were maintained within 3°, tibial varus/valgus angles were maintained within 1°, and posterior tibial slope was maintained below 7°, meeting the standards established by the aforementioned studies. This approach prolongs the life of the prosthesis and demonstrated the precision of the robotic implantation procedure.

Batailler et al. conducted a randomized controlled clinical trial comparing 80 patients who underwent robotic-assisted unicompartmental knee arthroplasty with 80 patients who underwent traditional unicompartmental knee arthroplasty from 2013 to 2017, with an average follow-up of 19.7 months. The results showed that the robotic unicompartmental knee arthroplasty group had better clinical outcomes and a lower rate of prosthesis loosening [31]. Similarly, Foissey et al. compared 197 cases of robotic-assisted monondylar replacement with 156 cases of patients in the traditional surgery group, with a mean follow-up of 61.3 ± 24.0 months, and evaluated related indicators such as lower extremity HKA angle, medial tibial tilt angle, posterior tibial flat prosthesis tilt angle, and joint line height, indicating that robotic-assisted monondylar replacement has its advantages. At the last follow-up, implant survival rates were higher in the robotic group [32]. In the past, the appropriate prosthesis type and good prosthesis position relied on preoperative 2D imaging and the surgeon’s surgical experience, which can lead to large errors in prosthesis size and position and inconsistencies in postoperative outcomes between different surgeons. The NATO Robotic-Assisted System is based on the pre-operative 3D reconstruction of the hip, knee, and ankle using thin-slice CT scans (< 1 mm) to define the relevant lines of force, osteotomy thickness, and prosthesis position, which is undoubtedly more intuitive and accurate. In the actual surgical procedure, we will adjust the osteotomy volume and prosthesis position according to the preoperative knee flexion/extension gap before the osteotomy as well as the prosthesis trajectory, and we usually reserve 2–3 mm of flexion/extension gap, which is sometimes difficult to be precise in traditional surgery, but easier to do in robotic surgery, reflecting the precision and consistency of the osteotomy and prosthesis position, which is key to the survival of the prosthesis.

Dunbar et al. [33]performed unicompartmental knee arthroplasty using the MAKO robotic system on 20 knees. The study required preoperative planning and postoperative imaging to demonstrate prosthesis placement error of less than 1.6 mm and varus/valgus alignment within 3°. Post-operative x-ray and CT results showed that the femoral prosthetic error averaged 0.8 mm and 0.9°, while the tibial prosthetic error averaged 0.9 mm and 1.7°, demonstrating the precision of the MAKO robotic system. Previous studies have mainly focused on imported robotic systems, while this study shows that the domestic Naton robot achieves satisfactory early results in terms of both prosthesis placement accuracy and patient functional improvement.

Kim et al. suggested that poor lower extremity power lines are also a common cause of revision, and we need to focus on correcting lower extremity power lines in robotic-assisted surgery at the same time [34]. We aim to restore the mechanical axis of the lower limb to neutral or near-neutral alignment. Thanks to the robotic system’s precise assessment of soft tissue tension, the surgeon can finely adjust the preoperative plan during surgery to accurately control the medial compartment gap, maintaining it within the ideal range of 2–3 mm throughout the full range of flexion and extension. This ensures that the contact trajectory between the femoral and tibial components remains in an optimal state. Additionally, with the real-time navigation provided by the robotic system, the surgeon can continuously monitor the lower limb alignment, avoiding excessive medial tightness and knee valgus. Traditional surgery relies heavily on preoperative full-length lower limb radiographs and the surgeon’s experience, whereas in robotic-assisted surgery, we simply follow the preoperatively planned alignment for osteotomy and implant positioning. This simplifies the reliance on subjective experience, enabling the restoration of normal knee kinematics and soft tissue balance, thereby improving postoperative range of motion and patient satisfaction.

Excessive intraoperative traction of the knee joint and repeated osteotomies may lead to soft tissue damage and hypertrophic scar formation within the knee, resulting in stiffness and pain during knee movement [35]. The precise navigation provided by the NATON robotic system eliminates the need for multiple osteotomies, thereby reducing trauma to the soft tissues surrounding the knee. This not only helps alleviate postoperative pain caused by soft tissue contracture but also minimizes scar tissue formation, enhancing the patient’s early postoperative mobility. Notably, in terms of safety, the precise navigation of the NATON robotic system prevents excessive bone resection. The protective mechanism of automatic power disconnection in the robotic arm ensures that critical vessels and nerves around the knee are not injured during surgery, significantly reducing surgical risks.

In a survival analysis of robotic versus non-robotic unicondylar replacement with up to three years of follow-up, St Mart et al. mentioned that unicondylar replacement with the Mako robot had a higher rate of early revision, mostly due to early infectious factors rather than prosthesis position [36]. In our study, no complications were found in any of the robotic surgery patients, although this was our first series of Nathon robotic surgery patients, so there is reason to believe that robotic surgery is safe.

The use of the Naton robot for unicompartmental knee arthroplasty involves a learning curve. In the early stages, the need for intraoperative registration and frequent calibration complicates the surgical process, resulting in an average operative time of approximately 100 min. However, as the surgical team becomes more familiar with robotic surgery, the average operative time can be reduced to approximately 50 min, a significant improvement. To optimize robotic surgery, it is advisable to develop a dedicated team that is thoroughly trained in the robotic surgical procedures and steps. This coordinated effort can further reduce surgical time [37]. In addition, regular maintenance and upkeep of the robotic hardware and software are essential, resulting in higher surgical costs compared to traditional methods. In addition, there are limitations in the compatibility of unicompartmental prosthesis designs from different manufacturers. Currently, the Naton robot is only registered for the Link fixed-bearing prosthesis and the Zhengtian fixed-bearing unicompartmental prosthesis, which limits the variety of prosthesis options available to patients.

## Conclusion

Short-term follow-up indicates a favorable prosthesis in situ rate for unicompartmental knee arthroplasty using the Naton robot, with satisfactory knee function and patient-reported outcomes.

### Limitations

Further verification of the long-term clinical follow-up results and prosthesis survival rates is needed. This study only conducted short-term follow-up due to various limitations, including limited resources and time, and did not conduct long-term efficacy follow-up. It was a single-center study with a small sample size, which may introduce bias. Future studies will extend the follow-up period and increase the sample size to further evaluate the clinical effects of Naton robotic-assisted unicompartmental knee arthroplasty, and provide valuable insights for its clinical application.

## Data Availability

The patient data will be made available from the corresponding author on a reasonable request.
